# The caring experiences of family caregivers for patients with advanced cancer in Uganda: A qualitative study

**DOI:** 10.1371/journal.pone.0293109

**Published:** 2023-10-25

**Authors:** Sarah Maria Najjuka, Angelique Iradukunda, Mark Mohan Kaggwa, Anastacia Nabyonga Sebbowa, Joy Mirembe, Kennedy Ndyamuhaki, Catherine Nakibuule, Joan Patience Atuhaire, Elizabeth Nabirye, Elizabeth Namukwaya, Sarah Kiguli

**Affiliations:** 1 College of Health Sciences, Makerere University, Kampala, Uganda; 2 Department of Psychiatry and Behavioural Neurosciences, McMaster University, Hamilton, Canada; 3 Forensic Psychiatry Program, St Joseph’s Healthcare Hamilton, Hamilton, Ontario, Canada; 4 Department of Palliative Care Medicine, College of Health Sciences Makerere University, Kampala, Uganda; 5 Department of Pediatrics and Child Health, College of Health Sciences Makerere University, Kampala, Uganda; Federal Ministry of Agriculture and Rural Development, NIGERIA

## Abstract

**Background:**

Cancer morbidity and mortality is rising in sub-Saharan Africa. Given this rise, family caregivers play an integral role in provision of quality cancer care services. This study explored the family caregivers (FCGs)/relatives’ experiences of caring for patients with advanced cancer (stage 3 or stage 4) in Uganda.

**Methods:**

This was a descriptive qualitative study exploring the lived experiences of FCGs of patients with advanced cancer attending care at the Uganda cancer institute. We purposively recruited twelve FCGs and conducted face-to-face in-depth interviews using an interviewer-guided semi-structured questionnaire. Data were analyzed by thematic analysis

**Results:**

The age range of participants was 19 to 49 years. Most participants were children of the patients (*n* = 7), had attained tertiary education (*n* = 7), and had taken care of their loved ones for at least one year (*n* = 10). Six themes emerged from data analysis; (i) caring roles, (ii) caring burdens, (iii) role conflict, (iv) health system tensions, (v) support and motivation, (vi) caring benefits, lessons and recommendations.

**Conclusion:**

Study findings highlight the fundamental role of FCGs in the care of their loved ones, and illuminate the neglected physical, psychological and social challenges of family caregivers amidst health system tensions and conflicting roles. The needs of family caregivers should be embedded within cancer care, prevention and control programs particularly in low resource settings.

## Introduction

Cancer is the leading cause of global mortality, accounting for nearly 10 million deaths in 2020 [1]. A significant rise in cancer related morbidity and mortality is being observed in low- and middle-income countries. In Africa; there was over one million new cases and 711,429 deaths in 2020, and a 70% rise in cancer cases has been predicted by 2030 [2, 3]. Similarly, Uganda registered approximately 34,008 new cases and 22,992 deaths in the same year (2020) [2]. Moreover, about 80% of newly diagnosed cancers are discovered when advanced, metastatic, and incurable [4].

Patients with advanced cancer (stage 3 or stage 4) suffer a significant burden of physical and psychological symptoms with pain and feeling drowsy/tired being the most prevalent [5]. Other symptoms include feeling nervous (57%), sadness (54%), and worrying (53%) [6]. This high symptom burden results in unplanned, prolonged hospitalizations and readmissions during which palliative care is the main modality of care [7, 8]. During this period, patients greatly depend on family caregivers (FCGs) who support their loved ones along the illness trajectory [9].

A systematic review by Zhu and colleagues highlights the unique nature FCGs’ experiences which are described as complex, multifaceted and challenging, associated with immense responsibility against the background of inadequate professional support [10]. At the time of diagnosis, FCGs accompany cancer patients to medical appointments, become the patients’ advocates, the primary decision makers on patients’ behalf, provide emotional support, and companionship, in addition to executing medical plans and helping patients with activities of daily living [11, 12]. Moreover, these roles are adopted suddenly with no prior preparation or experience and extend for several years until when the cancer is cured or takes the patient’s life [13].

Family caregivers face physical, social or emotional problems and responsibilities in isolation or in combination [14]. Physical effects include pain, fatigue, sleep disturbances, loss of appetite, and weight loss [15]. Family caregivers also report stress-related conditions such as depression, anxiety, insomnia, headache, and gastrointestinal symptoms [16]. The social impact of care giving includes financial difficulties, work/job interruptions, role changes, among others [17]. Spiritual concerns relate to reduced faith and hope, and drawing apart from God. Nevertheless, care giving has been described as a rewarding experience by some FCGs who report being satisfied for managing their care giving role, express happiness over quality time spent with their loved ones, ability to resolve issues and feelings of self-worth, and expression of love for the patient [18]. Despite the significant negative impact of care giving on FCGs’ wellbeing, FCGs often fall through the cracks of health care systems as their needs are frequently overlooked yet their distress tremendously affects patient outcomes [19, 20].

Ugandan FCGs are intensely involved in cancer care due to the high patient-to-nurse ratio (11,000:1) in the country [21, 22]. However, there is paucity of studies exploring the experiences FGCs of patients with advanced cancer in Uganda [22–25]. There are two multinational qualitative studies by different researchers with varying experience and interviewing skills which generates variability in the depth of interviews and one being a secondary analysis [24, 25]. Our study adopts a qualitative approach as a single team of experienced researchers to provide an in-depth understanding of FCGs experiences of caring for their loved ones with advanced cancer, exploring their roles, challenges, sources of motivation and support, and needs to inform interventions that support FCGs in their role.

## Methods

### Study design

This was a descriptive qualitative study among family caregivers (FCGs) of patients with advanced cancer (stage 3 or 4) at the Uganda Cancer Institute (UCI). We adapted a phenomenological approach that aims to describe the essence of a phenomenon by exploring it from the perspective of those who have experienced it, in terms of what was experienced and how it was experienced [26]. In this study, it allowed for an in-depth examination of lived experiences of FCGs for patients with advanced cancer [27].

### Study setting

The study was conducted at UCI, the only tertiary cancer treatment facility in Uganda. UCI provides both inpatient and outpatient services to Ugandans cancer patients and receives referrals from neighboring countries such as Kenya, Tanzania, Rwanda, South Sudan, Burundi, and the Democratic Republic of Congo [28]. UCI has a 1500 bed capacity, treating various malignancies in both adults and children [29]. Due to its location in the capital city of Uganda, most of the patients and their FCGs travel long distances to acquire tertiary cancer treatment from the facility, spending long periods of time away from their families. In addition, the UCI lacks proper accommodation facilities for FCGs who form an integral part of the health care provision particularly for patients with advanced cancer who are weak and require long hospital stay [22, 24].

### Sampling and recruitment

Eligibility criteria for FCGs to participate were; having a family relationship with the patient, aged at least 18 years, proficiency in English or Luganda and taking care of a patient with advanced cancer (stage3/4). We excluded caregivers who were physically ill or weak to complete the interview. We worked with clinicians at UCI to identify potential participants. The research team then approached potential participants and gave them information about the study; those interested were provided with an informed consent form and contacted two days later to schedule an interview. We purposively selected participants to include male and female, younger and older, various relationships to the patients and duration of care to ensure diversity of experiences of care. Participant recruitment continued until a point of thematic saturation was reached.

### Data collection

Face-to-face in-depth interviews were conducted to gather information from the participants between April and September in 2021. We used semi-structured interviews to enable reciprocity between the interviewer and the participant, allowing further exploration and or follow up by the interviewers and providing flexibility in discussions about potentially upsetting and sensitive questions [24]. The interview guide (S1 File) was designed by senior university lectures on the research team (SK and EN) with expert knowledge of qualitative research and vast experience with cancer care. It was based on a comprehensive literature review about FCGs’ experiences of cancer patients. [12, 24]. The tool was pre-tested prior to data collection to refine the content and enable researchers/data collectors to familiarize with the topics and flow of questioning. We probed information on their experiences prior to and during hospitalization of their loved ones. We also explored their roles, burdens, lessons learnt, and advice for possible improvement of cancer care services at the study site. Using a private room provided by UCI (to ensure participants privacy), we interviewed participants for about 45 to 60 minutes. We collected field notes, audio recordings, and memos during the interview.

### Ethical considerations

The Uganda Cancer Institute Research and Ethics Committee approved the study under registration number UCI-2020-11, and the Uganda Cancer Institute (UCI) administration provided permission to conduct the study. All participants provided written informed consent prior to enrollment in the study.

### Data analysis

Data collection and analysis were conducted concurrently. Transcribed audio recordings were made anonymous with a unique identification number following removal of personal identifiers. Four transcripts in the local language (Luganda) were translated and back-translated in to English by an independent translator before data analysis. Data coding was done manually using the inductive approach and data were analyzed using thematic analysis [30]. This involved the following steps: initially, transcripts were checked for accuracy and repeatedly read to familiarize one’s self with the data as line-by-line coding was done, identifying emerging ideas and themes within each transcript. Data were organized around key themes which were continuously reviewed, defined and named. Through this process, broader themes were developed to envelop the experiences of caregivers and create a picture of the data as a whole. A codebook was developed by three members of the research team (SMN, MMK and AI) who independently coded the transcripts using line by line coding. These team members met regularly to compare similarities and differences and to agree on the emerging patterns and anomalies across transcripts; ensuring consistency and reflexivity in the interpretation of the data. With the codebook in place, other team members were involved in the coding. Regular meetings were conducted with continuous discussions until reaching consensus about point of saturation and data was collected until there were no new emerging ideas. Analytic rigor was enhanced by searching for and resolving any contradicting cases in developing the themes. To ensure transferability of study findings, participants had maximum variation in care experiences, demographic characteristics, duration of caring, and type of relationship with their loved ones. A detailed record of sampling, recruitment, audio recordings and field notes, transcripts and the coding process was kept to provide an audit trail. We employed the Consolidated criteria for Reporting Qualitative research checklist (COREQ) to ensure comprehensive reporting [31].

## Results

### Sample characteristics

Saturation was reached at twelve in-depth semi-structured interviews involving an equal number of male and female family caregivers (FCGs) of patients with advanced cancer (stage 3 or 4). The participants’ age ranged from 19 to 49 years. Half of the participants were married and the majority (*n* = 7) had attained tertiary education. Participants were mostly children to the patients (*n* = 7) i.e., four sons and three daughters. Most (*n* = 10) of the participants had taken care of their patients for one year or more and had spent at least two weeks on the oncology ward. Details of the participants and their patient characteristics are presented in **Table 1.**

**Table 1:** Participant and patient characteristics (n = 12).

Participant characteristics								Patient characteristics			
Code	Age	Sex	Marital status	Education	Occupation	Care giver relationship	Duration of care	Age	Sex	Diagnosis	Duration on ward
01	49	M	Married	Tertiary	Teacher	Son	5years	6	M	Rhabdomyosarcoma	1week
02	19	F	Single	Secondary	Student (S.4)	Niece	2years	40	F	Breast cancer	5 days
03	36	F	Married	Primary	Housewife	Wife	2years	42	M	Lung cancer	2months
04	22	F	Single	Tertiary	Student	Daughter	6months	61	F	Skin cancer	2 days
05	32	M	Married	Primary	Peasant farmer	Father	1year	5	M	Acute lymphoblastic leukemia	6months
06	38	F	Divorced	Secondary	shop attendant	Mother	1 year	17	M	Burkitt’s lymphoma	2months
07	39	M	Married	Tertiary	Teacher	Father	10years	10	M	Brain cancer	3weeks
08	35	M	Married	Tertiary	Accountant	Son	2 years	78	M	Ca rectum	3 days
09	41	M	Married	Tertiary	Social-worker	Son	1 year	77	M	Ca Esophagus	2 weeks
10	23	F	Single	Tertiary	Student	Daughter	1 year	51	F	Cancer of the cervix	3months
11	19	F	Single	Secondary	Student	Daughter	3 months	48	F	Cancer of the uterus	1 month
12	38	M	Married	Tertiary	Nurse	Son	1 year	62	F	Cancer of the cervix	2 weeks

### Overview of themes

Six themes emerged from data analysis; caring roles, care giving challenges, role conflict, health system tensions, support and motivation, suggestions and lessons learnt. Each of these themes and their sub-themes are discussed in details below (**Table 2**).

**Table 2:** Experiences of caring for patients with advanced cancer.

Theme	Subthemes	Codes	Repetition of codes by participants
1.Caring roles	Support with activities of daily living	(i). Bathing the patient	P2*1/P3*1/P4*1/P9*1/P12*3
		(ii). Feeding the patient	P1*2/P2*1/P3*2/P5*2/P7*1/P9*4/P11*4/P12*1
		(iii). Helping the patient to ambulate	P4*2/P8*2/P9*3
		(iv). Washing patients’ linen	P1*2/P3*/P3*1/P7*2/P9*1/P10*3
	Coordination of care	(i). Communication of the patient’s concerns to health workers	P1*2/P5*1/P7*1/P8*1/P9*1/P10*1/P12*1
		(ii). Taking the patient to and from the hospital	P1*1/P2*3/P3*2/P4*2/P5*1/P6*3/P7*4/P8*2/P9*2/P10*1/P11*1/p12*1
		(iii). Drug administration	P1*2/P2*1/P6*1/P12*1
		(iv). Follow-up of investigations	P3*1/P5*2/P6*1/P7*2/P8*1/P9*2/P11*1/P12*1
	Decision making	(i). Decision to seek medical care	P1*1/P2*1/P4*1/P6*1/P9*1/P12*1
		(ii). Decision to seek specialized care	P4*1/P5*1/P6*1/P9*1
2.Caring burdens	Financial drain	(i). Buying expensive drugs	P1*1/P3*2/P4*2/P6*2/P8*1/P9*2/P10*1/P11*1
		(ii). High transport costs	P*1/P1*1/P5*2/P7*3/P8*1/P10*1/P11*1/P12*1
		(iii). Very costly investigations	P4*1/P5*1/P7*2/P9*1
		(iv). High feeding expenses	P1*1/P3*1/P4*1/P5*1/P6*2/P7*1/P9*1/P10*1/P11*2
	Physical problems	(i). Headache	P2*1/P9*1
		(ii). Chest pain	P2*1
		(iii) Fatigue	P1*1/P3*2/P4*1/P6*1/P11*1
		(IV) Body pain	P4*2
		(V) Sleep disturbances	P3*2/P4*1/ P5*1/P8*1/P9*1/P11*1
	Psychological distress	(i). stress	P2*2/P3*1/P4*1/P6*1/P11*2
		(ii) Fear	P1*2/P2*1/P3*1/P4*1/P9*1/P11*4
		(iii) Sadness	P1*1/P2*1/P4*4/P5*1/P10*1/P12*2
		(iv). Worry	P1*2/P2*1/P4*1/P5*1.P6*1/P7*1/P8*1
		(v). Helplessness	P3*1
	Social challenges	(i). Lack of time for friends/other loved ones	P2*1/P3*1/P10*1
		(ii). Misunderstandings within the family	P4*2/P7*1/P8*2/P10*2
	Spiritual/Cultural challenges	(ii). Lack of time to go to church	P2*1/P4*1/P10*1/P11*1
		(i). Difficulty in bathing the patient of opposite sex	P4*1/P9*1P10*1
		(ii). Use of alternative medicine	P1*1/P5*3/P10*1
		(iii). Negative cultural beliefs	P5*1/P9*1
3.Role conflict	Failure in employment and educational roles	(i). inability to go the work place	P1*1/P5*2/P6*1/P7*1/P9*2/P10*1
		(ii). Complicated relationship with employers	P6*2/P7*1/P8*1/P12*1
		(iii). Loss of employment/Property	P1*1/P5*1/P9*1//P10*1
		(iv). Absence from school	P9*1/P11*1
	Inability to balance caring roles and family responsibilities	(i). Lack of time to visit the family	P3*1/P9*1/P11*1
		(ii). Inability to execute family roles and responsibilities	P2*1/P3*2/P4*1/P5*3/P6*1/P7*6/P9*1
4.Health system tensions	Admission process	(i).) Delay to make a diagnosis	P2*1/P3*2/P4*2/P5*2/P8*1/P9*1/P10*1
		(ii).Prolonged/complicated admission process	P7*1/P8*2/P9*2/P10*1/P11*1/P12*1
	Hospital environment	(!). Lack of proper accommodation for caregivers	P1*1/P4*2/P5*1/P10*1/P11*3
		(ii). Unclean hospital Lavatories	P1*3/P5*1/P10*1/P11*1
		(iii). Overcrowding in the wards	P2*1/P3*1/P11*1
		(iv). Insecurity around the facility	P3*1/P9*1
	Health professionals	(i) Kind health workers	P1*1/P4*2/P6*1/P7*4/P9*1/P10*1/P11*1
		(ii). Good communication	P4*1/P7*1/P9*1
		(iv) Few health workers	P1*1/P7*1/P8*1
		(v). Caregivers’ neglect	P5*1/P8*1
		(vi). Poor attitude	P1*2/P3*2/P4*1/P5*4/P7*2/
		(vii).Lack of/Poor communication	P1*2/P2*1/P4*2/P5*2/P7*2/P8*1/P9*1
5.Support and motivation	Sources of support and motivations	(i). Peer support from other caregivers	P1*2/P5*1/P7*1/P8*1/P11*1
		(ii). God as a source of motivation	P1*1/P3*1/P4*1/P6*1/P7*3/P8*1/P11*1/P12*2
		(iii) Improvement in the patient’s condition	P1*1/P2*1/P3*2P5*2/PP6*2/P7*1/P10*1/P11*1
		(iv). Love for the patient(desire to give back)	P2*1/P3*1/P5*1P8*3/P9*1/P10*2
		(iv). Support from the spouse/family/friends	P1*2/P2*1/P3*3/P4*2/P5*2/P6*2/P7*1/P8*3/P9*3/P10*1/P11*1/P12*2
		(vi). Support from the community (church members, politicians, Non-governmental organizations)	P2*1/P5*1/P6*1/P9*1/P10*1/P12*1
		(vii). Support from the hospital system/administration	P5*2/P10*1/P11*1
	Support needs (Needs of caregivers)	(i). Financial support	P4*1/P7*1/P11*1
		(ii). Psychological support	P8*1
		(iii). Conducive hospital environment	P11*1
6) Caring benefits, lessons and recommendations	Social benefits	(i). increased bond between caregiver and patient	P2*4/P8*4/P9*1/P10*1/P11*1
		(ii). Unity within the family	P1*1/P3*4*P4*2/P4*2/P6*1/P7*3/P8*2/P9*1/P10*2/P11*1/P12*1
		(iii). Acquiring new friends from the hospital	P1*1/P5*1/P8*1/P11*1
	Spiritual and religious benefits	(i). improved relationship with God	P1*2/P2*1/P3*2/P4*2/P5*1/P6*3/P7*3/P8*3/P9*2/P10*1/P11*2/P12*3
	Other benefits	(i). Enhanced cancer and cancer prevention knowledge	P1*1/P4*1/P5*1/P7*1/P8*1/P10*2/P11*2
		(iv). Respect of humanity	P4*1/P5*1/P7*3/P8*1
		(v). Minor hospital procedures such as drug administration and wound care	P2*1/P6*1

### Theme 01: Caring roles

Care giving was described as a long journey involving travelling long distances to the tertiary cancer facility when escorting patients to multiple hospital appointments, being with patients in hospitals during prolonged periods of hospitalization which are characterized by episodes of patient improvement and recurrence of symptoms as cancer became advanced. FCGs described their multiple roles in patient care such as; support with activities of daily living, coordination of care, decision making and offering nursing and practical care. There was no variation in the caring roles with age, gender, relationship with patient or duration of care among family caregivers.

#### Subtheme 1: Support with activities of daily living

Family caregivers provided support to their loved ones with Activities of Daily living (ADLs), for example, bathing, dressing, washing patients’ clothes, and feeding them; a full-time responsibility. This is mainly because majority of the patients were weak and unable to perform ADLs due to the severity of advanced cancer, which made them dependent on their FCGs. FCGs also provided a clean, comfortable, relaxed, and calm environment for patients.

*“I wake up*, *if he wants to eat*, *I get him breakfast*, *then I see what he wants to eat for lunch*. *Sometimes I prepare juice*. *I wash*, *I bathe him when he is weak*, *when he is unable to move*, *and we use a bucket*.*”* ***(*Participant 3)**

*“Early in the morning*, *when Mzei [my father] has woken up*, *…I clean him in the mouth and then the body*, *and because he cannot move out*, *I dress him from here*, *so I have to remove the pumpers and change him into clean ones*. *Now when he is clean*, *I lay the bed …silence*… *after that*, *I then start to look for what to feed him*, *…*. *I feed him*. *Sometimes I massage him to make him feel relaxed*. *After I give him his medication then I wash his clothes*.*”* (**Participant 9**)

#### Subtheme 2: Coordination of care

Family caregivers were involved in coordinating all aspects of patient care. Starting from the time the patient experienced their first symptom, FCGs; especially those who were parents were involved in seeking health care services, coordinating the family, health care providers, religious leaders, and the patient by carrying out patients’ tasks, and ensuring execution of all needed patient tasks. FCGs interacted with health care providers through the following ways: communicating patient’s conditions to the health care providers, waiting for patients’ results from the laboratory or radiology department, taking samples to the laboratory, and executing the doctors’ prescriptions such as making sure patients do exercises, take their medications, among others. In addition, if the patient’s condition deteriorated, they would call the health care providers for emergency care provision as demonstrated in the following quotations.

*“My day is organized depending on doctors’ ward round schedule because very early in the morning they ask me how he is and how he slept*, *and I tell them what has happened and they note it*.*”* **(Participant 1)**

*“One of the major activities I do are*: *to move Mzei around*, *if he is supposed to go to another ward*, *or at the OPD*, *I have to take him on a wheel chair and I move him around*. *When it is time for waiting for results*, *I have to make sure that I keep around to get the results*. *When we have been told to buy drugs*, *which are not inside here [UCI]*, *it’s me who moves out to buy the drugs for him*.*”* **(Participant (9)**

#### Subtheme 3: Decision making

Family caregivers were also pivotal in making critical health decisions that related to the care of their loved ones especially for elderly patients and children. Such decisions included: decision to seek medical care when symptoms first presented, decision to seek advanced/ specialized care when symptoms persisted or worsened, decision to seek for a second opinion when not contented with the diagnosis, requesting for referral to other facilities to get better health care for the patients, requesting for discharge due to various reasons such as failure to afford care or services, among others.

*“…*. *I made a decision after 2 months in the clinic with no change… I said … let me look for another way forward*. *Then I went to the hospital and found a senior doctor*, *he examined the swelling and then used an Ultrasound scan*. *He later told me that I needed to come to national referral hospital for further management and I decided to bring my child to this hospital*.*” (***Participant 5**)

*“When we went back home*, *he was no longer swallowing food*, *even swallowing water or juice became too difficult*, *so we decided to organize and we brought mzei back to this hospital and we met a Doctor who told us that since mzei could no longer feed through the mouth*, *they needed to put a tube for him to feed”* **(Participant 9)**

*“…about 6months ago*, *I went to visit her and in our conversation*, *she told me that she was bleeding from her private parts*. *So I thought that we needed to take her to the hospital for investigation*. *So we went to the hospital near home and they asked us to do a scan and then later we were advised to do a biopsy*.*”* **(Participant 12)**

### Theme 02: Caring burdens

Care giving negatively impacted FCGs’ wellbeing in multifaceted ways including financial, physical, psychological, social, spiritual, and cultural.

#### Subtheme 1: Financial drain

A cancer diagnosis often presents financial difficulties for patients and their families particularly when the person diagnosed with cancer was the breadwinner and when the caregiver has to leave gainful employment to take on the care giving role [32]. Due to limited cancer services in rural areas where majority of the cancer patients live, patients had to obtain cancer care from UCI which involved long distance travels and high transport costs. In addition, despite the fact that most health services were free at the UCI, FCGs were faced with high costs of food, buying the unavailable medicines, and paying for some patients’ investigations that were not available at UCI. Caregivers who had cared for their patients for a longer duration (one year or longer) expressed more financial burden of care giving as demonstrated in the following quotations.

*“Feeding is very expensive especially for us who come from very far with no other source of income*, *no place to work; the hospital can’t provide all your needs*.*”* (**Participant 5**)

*“The transport is difficult because we use public transport; we use a taxi from the village to this side [UCI]*. *Transport fee is too much and the taxi passes on a bad road*.*”* (**Participant 10)**

*“The cost of these investigations is far beyond what the local people can afford*. *It is like the hospital is for a certain class of people…*. *for me I thought that services have to be provided to the local person but when you come here…*. *it is your cost*. *If you are to get any service*, *they first assess your affordability and sometimes if you can’t*, *they send you to some other place but not where you expected to be*.*”* (**Participant 7**)

In order to provide the best care for their loved ones amidst financial constraints, some caregivers reported to use up all their personal savings while others sold their property such as land, domestic animals and other personal belongings.

*“I had a financial package which I was given as my contract was ending… so right now*, *I have used all of it and I had some good harvest of maize as of last year’s second season*, *so that’s what am now selling*. *I also sold my cow to able to take care of my father*.*” (***Participant 9**)

*“Mum had a plot of land*, *we sold it; it is what has been keeping us here all this time”*. *(***Participant 4**)

#### Subtheme 2: Physical problems

Family caregivers often focus on the patient’s needs and ignore their own health needs resulting in their ill-health. Many FGCs reported a variety of health problems that resulted from long-term care giving roles. Physical problems mentioned mostly fatigue, sleep disturbances, headaches and body pain. These physical problems were mostly reported by FCGs who had cared for their loved ones for a two years’ duration, they said,

*“It is tiresome but I am not taken up because I know why am doing all this*. *I get physically exhausted but I do not get disgusted with the whole care giving experience*. *Sometimes the journey the pharmacy outside the hospital is long*… *that can be tiring*.*”* ***(*Participant 3)**

*“At times if the patient is badly off*, *you can’t sleep*. *Throughout the night*, *you may rest for only 2 hours*.*”* **(Participant 2)**

#### Subtheme 3: Psychological distress

Caring for a patient with advanced cancer may lead to psychological distress among FCGs. Besides, lack of preparation for a demanding role of a caregiver coupled with fear of death of a loved one creates a state of shock and sadness among FCGs. Many FCGs reported constant sadness, worry, stress, and feeling of helplessness especially when the patient’s condition worsened or deteriorated. Participants expressed these feelings in the following quotations.

*“It reaches a time when I feel unwell*. *When I see the situation that my mother passes through*, *when she cannot walk*, *there is a time when she cannot feed herself*, *so it has affected me so much because I feel sad*. *…especially*, *when I see my mother*, *who produced me and took care of me*, *in this very bad situation*. *I really feel stressed and worried*. *(***Participant10**)

The distress manifested with somatic symptoms such as headache and chest pain in some of the FCGs.

*“I don’t feel all that good because she is unwell*. *I feel chest pain and sometimes when I think a lot*, *like when you are seated and start thinking*, *what if my aunt dies*, *you are always worried*. *I even get headache*. (**Participant 2**)

#### Subtheme 4: Social challenges

The care giving role was described as lengthy and time-consuming requiring full participation of FCGs. Participants reported lack of time to attend social events, visiting friends and relatives.

“*I don’t have time with my friends*, *now like this time am with her*, *comforting her*.*”* (**Participant 2)**

Unfortunately, FCGs also cited misunderstandings with family and other social support members such as friends brought about by the caring role. Disagreements were reported among spouses, siblings, friends, and children especially due to failure to share collective responsibility to take care of their patient. This care giving responsibility led to many family disagreements to the extent of causing patient neglect as seen in the following quotation.

*“If someone [relative] sends money*, *they think that they have assisted with care giving… fine*, *it helps*, *but it is not similar to you who is there all the time*. *They [relatives] were thinking that care giving is a walkover*, *so I also got annoyed as a normal person*, *and I said*, *fine*, *our father is there; you also help him*. *I left everything to them*. *I told them*, *from today*, *don’t even call me because I have left him for you*. *Get your own car*, *I have suspended my car because they had reached at the level of thinking that the car is for daddy*. *So*, *I left him to them*. *Last Friday they had given up and said that they don’t have money*.*”* **(Participant 8)**

#### Subtheme 5: Spiritual challenges

A few participants reported having no time to express their spirituality or engaging with God through going to church, fasting, and having more prayers; a situation that made them distressed and sad as elaborated by this caregiver.

“*I am not praying now as I was before*, *but I usually do when I am going to eat or sleep*. *I cannot move away from home to go to church because I cannot leave the patient*, *and I go for prayers*. *I really feel bad because I was used to going to church*.*”* (**Participant 10**)

*“Yes it (care giving) has affected me because I no longer get time to go to church on Sunday*.*”* (Participant 11)

#### Subtheme 5: Cultural challenges

Family caregivers struggled with communities’ cultural beliefs which negatively influenced their patients. For instance, some communities had strongly held cultural beliefs which birthed misconceptions that cancer was a mysterious disease caused by witchcraft, hence the use of alternative medicine such as local herbs to alleviate symptoms of advanced cancer. This delayed the cancer diagnosis, proper treatment, and increased patient suffering.

*“They started telling mummy that she might be bewitched*, *you can go to witch doctors or you can use herbs*. *And many people were giving her herbal medicine to take but the situation did not change*. *So*, *it worsened and reached the extent of needing blood transfusion*.*”* (**Participant 10**)

Many caregivers described bathing a parent or an elder as immoral, and traditionally unacceptable and this was especially taboo if the parent was of opposite sex. However, being the only alternative to helping the patient, they did it despite the difficulty.

*“Bathing my mother bothers me*, *but she is my mother*. *My culture does not allow bathing a parent*, *it is even worse if it is an opposite sex but for a girl it is somehow acceptable*. *In our case even if I were a boy*, *I would still do it*.*”* (**Participant 4**)

*“Most challenging is cleaning and dressing the mzei*. *In our tradition*, *being a father*, *there are areas on the body that you shouldn’t see*, *but now because of the sickness*, *I have to keep the Mzei clean*, *you really have to do it but at the back of your mind*, *you feel oh no*! *traditionally am going beyond…”* (**Participant 9**)

### Theme 03: Role conflict

Majority of the FCGs were never prepared for the care giving role. The care giving role for most FCGs was added onto the many other essential pre-existing roles/responsibilities they had in their lives and it became difficult to balance the multiple roles leading to prioritization of the care giving role which often resulted in the failure to maintain previous roles.

#### Subtheme 1: Failure in employment and educational roles

Employers, supervisors, academic institutions, and lectures/teachers fail to understand the caregivers’ situation and expect them to execute their usual roles/responsibilities. This presents a role dilemma where FCGs have to choose between their pre-existing roles/responsibilities and taking care of their loved ones. Most FCGs chose caring for the patients, losing their jobs and dropping out of school for student FCGs.

*“I have to go to work*, *but when the patients’ condition worsens*. *It is painful for me to leave him alone*. *I have to go and work; you know the bosses[employers] don’t mind or care about anything*. *They want me also to work*. *They don’t mind about my child*. *Sometimes I feel like I should leave work because of my child*, *but if I leave*, *we cannot survive here [at the hospital]*.*”* (**Participant 6**)

Family caregivers who were also employers failed to supervise their businesses resulting in losses.

*“I have failed to monitor my salon*, *people working there tell me that customers are not there but my friends tell me those people are making more money but the money they give me at the end of the day is too little*.***”* (Participant 1)**

#### Subtheme 2: Inability to balance caring roles and family responsibilities

Family caregivers mentioned failure to fulfill their family responsibilities. Care giving roles take away family heads and leads to family neglect. Receiving care in a hospital far away from their homes made it hard for them to perform their family roles, thus leaving young children unsupervised and having no primary providers. Family caregivers reported losing family property, feeling despised for inability to take care of their families, and failure to take their children back to school. Role conflict between care giving for other family members and the patient is an additional challenge.

*“Since am a peasant farmer*, *I stopped cultivating land; I lost my garden and animals*. *When you are not around*, *no one can take care of your property like you*. *So*, *when I went back home*, *they told me that my cows were stolen*.*” (***Participant 5**)

*“…as the head of the family*, *when you see what you are supposed to do being done by someone else*, *you become irritated and sometimes that person does certain things without telling you*, *you feel despised*.*”* (**Participant 7**)

### Theme 04: Health system tensions

This study was conducted at a national referral cancer facility that received patients from all over the country and neighboring countries. As result, the facility offered care to large numbers of patients which often overwhelmed the facility’s health care system. Long waiting time, inadequate hospital facilities, and inadequate attention from health workers were the main challenges highlighted by participants.

#### Subtheme 1: Long waiting time

Family caregivers demonstrated delay in making a cancer diagnosis and described the admission process at the tertiary facility as complicated and prolonged. They expressed concern with long waiting time between the time of referral and the doctor’s appointment at the referral facility. Some reported being worried that the patient’s condition could have worsened before being attended to. Caregivers would become desperate during this period and would consider alternatives such as seeking care from the neighboring countries, use of alternative forms of treatment, and trying spiritual interventions.

*“At booking*, *the waiting period is too long for the sick person*, *you find someone is so sick but they are giving him a long period of time to come in order to make a treatment plan*. *If you go up for booking to see a senior health professional*, *it can take you like three months before you see them*.*”* (**Participant 8**)

*“When we were given an appointment date in September*, *we got many thoughts*, *we were afraid that by the time the date reaches*, *the disease will have escalated*. *We thought that the immediate solution was to go to another country so that we can get the first treatment*. *Until when my father decided to wait*, *we were planning to go to Kenya for an urgent solution*.*” (***Participant 8)**

*“…it’s not straight forward*, *because when we came*, *first we had to go through the process*, *to make a file*, *they gave us a bed*, *it took two weeks time to see the senior doctor and yet she wasn’t feeling well*, *so we couldn’t go back home in two weeks*.*” (****Participant* 12**)

#### Subtheme 2: Overcrowded, unsecure, non-sanitary hospital facilities

Family caregivers described the in-patient wards as being overcrowded, lacking privacy as both male and female patients with their FCGs shared the same wards. Due to lack of enough hospital beds, some patients were left lying on the floor, which increased the risk of hospital acquired infections for these vulnerable caregivers. Some beds had very old mackintosh covers that were not water proof and were often reused by various patients; further allowing body fluids from patients to seep through.

*“We experience body pain because we sleep on the floor*. *The body becomes weak and tired*. *Because of this*, *one can even fall sick yet they are ideally the caretakers”*. *(***Participant 4**)

*“…*. *some other mattresses there have now become old and if a patient using the bed urinates*, *it will pass through the mattress*. *The mattress then absorbs the urine*. *Then another person will use the same mattress when the other patient is discharged or dead*.*”* (**Participant 5**)

*“… it is about the beds*, *that is where patients get difficulties*, *you come and find when beds are over and they tell you lay down*. *It is very easy to get infections because some people pass by you when they are from the bath room; they step on the beddings so it’s easy to get an infection*.*” (***Participant 10)**

The lack of sleeping facilities for FCGs was also reported, as many slept on the hospital floor, verandas, and some shared a bed with their patients. This could have resulted in the increased fatigue and body pain reported by FCGs. One caregiver reported being worried that something dangerous could bite her while sleeping in the grass (outside).

*“We don’t sleep well*, *some of us sleep outside*, *when rain comes*, *it’s too cold*. *We sleep on boxes*. *We experience body pain yet we have to take care of patients*.*”* (**Participant 4**)

*“Because we sleep outside*, *something dangerous can come*, *I fear snakes so when am outside I fear… because somebody can even use a stone to hit me*.*” (***Participant 11**)

Furthermore, the toilet/bathroom facilities were reported as dirty and lacking privacy since they were being shared by patients and FCGs of different gender. Caregivers were afraid of acquiring infections from such facilities.

*“…*. *about the cleanliness of the bathrooms*, *the bathrooms we have inside the ward are dirty which is easy to come when you’re not infected and you get sick*, *or if you have an infection and end up getting another infection*.*”* (**Participant 10**)

Finally, caregivers reported occasions of insecurity within the health facility which often lead to theft and loss property such as money, patients’ hospital records and other personal belongings.

*“When we were at the hospital*, *we were robbed*. *A bag with our money*, *phones*, *and medical cards was stolen*.*”* (**Participant 3)**

*“… unfortunately*, *on the 15*^*th*^ *when the CT scan was supposed to be done at the emergency department*, *early in the morning*, *my bag which had my property and the personal file which contained the appointment letter to do the CT scan and to meet the doctor was stolen*, *so meaning that I had to start tracing the file*, *which meant scheduling another appointment*.*” (***Participant 9**)

#### Subtheme 3: Experience with health professionals

Some FCGs were appreciative of health workers’ efforts while treating their loved ones. They described them as being kind, friendly and responding promptly when called to help the patient or FCGs. Health workers were also appreciated for providing information and explaining the patient’s condition to caregivers, making them confident in uncertain situations.

*“Many times*, *most especially the senior doctors*, *whenever they are going to do anything*, *we go through the discussions on what to expect out of whatever they are doing at any time*.*… you know that anything can happen so when they let me know*, *I am also confident*.*” (***Participant 7**)

*“The doctors have not treated us badly*, *they have treated us well*. *They have given mummy all the blood she needed*. *they have given her medicine and even the way they talkto us*, *they have not been difficult for us*. *You go and consult him or her and they reply to you very well*.*”* (**Participant 10**)

On the contrary, other FCGs reported that health workers were few to attend to the many patients on the ward and some were rude and unapproachable, and had no time to listen to patients and caregivers’ concerns.

*“One time a caretaker called a nurse because patient was badly off*. *They unnecessarily shouted at her*. *Eventually that patient even died*.*”* **(Participant 3)**

*“You find that the doctors are too busy to listen to some patient’s and caregivers’ concerns*. *They don’t have time*. *It is only (yes or no)*, *and that is so negative*. *The patients and caregivers are left in the blue*.*” (***Participant 7**)

In addition, participants mentioned being ignored by health workers and the health system which focus on only patients, yet FCGs also require their attention.

“…but more so now on the side of doctors and health workers, they only put their attention on the sick person, not knowing that the caregiver may be going through a hard situation, which is not good.” (**Participant 8**)

### Theme 05: Support and motivation

In order to perform their role efficiently, FCGs need support in all aspects of their caring role. In this study, participants described their sources of support, motivation, and areas in which they needed more support. These are summarized in two subthemes below.

#### Subtheme 1: Sources of support and motivation

Caregivers reported obtaining mainly financial and emotional support from their relatives, friends and church members who often visited and prayed for them while in the hospital. The health system also had a waiver system that supported FCGs to pay for unaffordable investigations.

*“… I have family members*, *church members and friends who are helping me*. *Church members have come home*, *like three or four times*. *They come as a group*, *and they sit with us*, *whatever they have*, *they give it to us*. *Also*, *our friends who are sending us money… those with money come at home and give us what they have*.*”* (**Participant 9**)

*“After finishing the first dose*, *I was supposed to repeat the same investigations for the child*. *That day*, *I went to the executive director’s office*, *which helped me and I paid only 150*,*000shs*. *That money actually came from my friends*.*”* (**Participant 5**)

Sources of motivation and hope to continue caring amidst challenges included; the love that FCGs had for their patients as they wanted to see them improve, observing their patient and other patients with a similar illness improve, the feeling they had to give back to their loved ones who took care of them before the illness, support from other caregivers, and the hope to be rewarded by God.

*“What always motivates me to give in totally for the patient is the positive response to treatment*. *It is really encouraging*. *It overshadows all other problems that you are going through*, *when you see your patient responding in comparison to how you began*.*”* (**Participant 7**)

*“I have no problem with taking care of him because this man has really been there for me*. *Personally*, *my husband died when I was still young*. *He has been the one taking care of my children and me*...*Even giving them school fees*. *I don’t see any other way I can repay him*.*”* (**Participant 3**)

#### Subtheme 2: Support needs

Financial support was the major form of support needed by participants in this study. Others included support in carrying out day-to-day activities such as bathing, lifting/turning patients, and patient ambulation.

*“Where I have reached now*, *I need financial support because we are remaining with about 2 cycles of treatment but it involves a lot of money which we have to struggle to get…*. *especially now we are in the sixth cycle and we have to do other investigations*, *you find that the investigation costs are always high yet they will not only need one*, *they will need several of them*.*”* (**Participant 7**)

*“I need support carrying my sick mom*. *Maybe if we were two people*, *carrying her would be easier*.*”* (**Participant 4**)

### Theme 06: Caring benefits, lessons and recommendations

Family caregivers described care giving as a rewarding experience especially for FCGs who felt they performed their role well. Caring benefits were mainly embedded in the social and spiritual domains. Many FCGs described a sense of family unity within family members often visited their loved ones while in the hospital and offered financial and emotional support to both the patient and the caregiver. FCGs also reported acquiring new friends (FCGs for other patients) since they stayed in the hospital for a long time.

“*I have got new friends from the hospital*. *Also*, *it [caring for the patient] has united us as a family*, *like mum being sick has created a good relationship within the family*. *There are relatives from mum’s side*, *I was telling them to come and visit us*. *… They came as a group; we were so happy as a family*. *It has also improved the relationship between us as the children at home because they all gather and come to take care of mum because in the past*, *we were distant*, *but it has united us as a family that makes me so happy*.*”* (**Participant 10**)

*“…but overall I would say*, *that her illness has brought us together as a family*, *we are working as a team because the demands are so many*, *even those relatives who used not be to in touch can now call us to know about her condition*, *it would be harder if I was the only one doing this*.*”* **(Participant 12)**

Almost all participants appreciated the impact of the care giving role on bringing them closer to God. Spirituality was seen as a means of seeking for divine power to help relieve their current stressors, for example, praying for miracles to cure their loved ones, solving their financial problems, reducing patients’ symptoms, among others. To many, praying (spirituality) was the coping strategy used in cases of stress.

*“I believe that God is present and he is taking care of my mother*.*”* (Participant 3)

*My only hope is for God to answer my prayers*. *Every day I fast for my son and I pray every morning and night time*. *I pray for him*. *I say…(God it is you to heal my child because you are the most doctor*, *you are one that created him) and I pray for him like that*.” (**Participant 6**)

*“I just pray to God for her life and I hope she will be better*.*”* **(Participant 12)**

Participants also mentioned learning one thing or another from their caring experience. These included: knowledge about cancer i.e., its signs and symptoms, risk factors, prevention, and treatment modalities; proper feeding practices, handling difficult life situations, treating other people well and helping those in need, performing simple medical procedures like wound care, removing intravenous lines, administering medications, among others. Some of the lessons are demonstrated by the following quotes.

*“It has taught me how to share and to give a hand where possible*, *however small it is*, *it can be very important and vital*. *Caring about others is important because we are all prone to vulnerability and you can’t know what might happen tomorrow*.*”* (**Participant 7**)

*“I have learnt to give medicines*, *like sometimes when the nurse is not around and water (Intravenous drip) gets finished*, *I just remove it by myself*.*”* (**Participant 2**)

*“I learnt how to wash wounds*, *to clean him in time*, *to change*. *Most especially I learnt how to perform proper wound care*.*”* (**Participant 6**)

Family caregivers suggested various ways to improve their experiences of caring for patients with advanced cancer. These ranged from hospital-based reforms to the entire health system in the country. They wished that all services such as investigations, drugs and medical procedures were available or performed within the same hospital (UCI) to avoid moving weak and fragile patients up and down. In addition, FCGs suggested that they needed to be supported by psychologists as they were at high risk for psychological problems which in turn negatively affects patients. FCGs also proposed that the government should decentralize cancer care and provide financial support to FCG/patients particularly to buy medicines for their patients.

*“… how I wish all the services were within here*, *because now Mzei is weak*, *but you have to take him down*, *or up*, *he comes back with almost no energy*. *We were up and down*, *we came back on a motor cycle and Mzei almost collapsed*, *so how wish all these services were just within this hospital such that we don’t have to move a lot of distances*, *sometimes you have to call for an ambulance*, *and the ambulance not for free*.*”* (**Participant 9**)

*“According to my personal observation caregivers need psychologists to talk to them because I have seen on several occasions*, *like yesterday when I was on the ward*, *a caregiver slapped a sick person due to frustration*.*”* (**Participant 8**)

*“… the government can support caretakers by giving them money when a person is sick*. *Money for buying drugs*. *Like some medicine that the government can’t provide*, *you have to buy them outside the hospital*. *Those ones are very expensive*.*”* (**Participant 5**)

## Discussion

This study explored the family caregivers’ (FCG) lived experiences of caring for patients with advanced cancer (stage 3 or 4), receiving care at Uganda Cancer Institute (UCI)—a national referral cancer care facility in Uganda. We identified six themes: (i) caring roles, (ii) caring challenges, (iii) role conflict, (iv) health system tensions, (v) support and motivation, and (vi) caring benefits, lessons and recommendations.

Family caregivers described care giving as a full-time job that stretches from the time when the patient experiences their initial symptoms throughout the illness trajectory, during which they support patients with activities of daily living, decision making, and coordination of patient’s care. This finding supports earlier evidence that recognizes FCGs as crucial and primary supporters of patients with advanced cancer throughout the course of the illness [22, 24]. Besides, the underfunding of health care systems in low- and middle-income countries results in poor hospital staffing with bedside nurses which makes FCG a major human resource to fill this gap [33]. Taking care of a patient with a terminal illness is time consuming and physically demanding [34]. Therefore, it is not surprising that FCGs experienced role conflict with failure to balance their caring roles with job, education and family responsibilities. One study in Nairobi also emphasizes role conflict as one of the frequent challenges FCGs face as they take care of their loved ones [35].

Participants described experiences of physical symptoms such as fatigue, body pain, sleep disruption, among others, similar to findings from other studies in Uganda and elsewhere [24, 36].

Family caregivers in this study site lacked proper accommodation and end up sleeping on the hospital floor, verandas and hospital walk-ways. This, in part explains their body pains and sleep disturbances. Despite the fact the hospital/government has put in place accommodation facilities for FCGs, it is not enough to accommodate FCGs from all over the country. Strategies to decentralize specialized cancer care could mitigate this problem by reducing patient numbers at the referral cancer facility.

Participants also reported psychological distress with frequent symptoms of depression such as sadness, constant worry and sleep disturbances associated with their care giving role; a finding that is consistence with other studies. For instance, an updated systematic review and Meta analysis published in 2022 found the pooled prevalence of depression among cancer care givers at 42.2% for cross sectional studies and 33.6% for longitudinal studies [37]. In Uganda, one study by Simpson and colleagues describes the prevalence of depression among cancer caregivers at 8.2% [23]. Depression among FCGs could be explained by the fact that providing care to a family member with terminal illness comes uncertainty of the future and fear of loss of a loved one which could increase mental health symptoms among FCGs. Unfortunately, care giving also deprived FCGs potential coping mechanisms and sources of motivation such as inability to go to church for prayers attributed to time limitation, and disorganizing relationships. This could have worsened their psychological distress.

The financial burden of care giving was a major finding in this study especially for FCGs who had taken care of their loved ones for longer durations (at least one year). Participants reported having financial difficulties with high hospital costs in terms of medicines and investigations, feeding and transport costs that some FCGs resorted to selling their property and using up all their savings in order to cope with these demands. Most of the cancer patients and FCGs in this study travelled long distances to reach the national referral cancer facility, hence high transport costs. In addition, the hospital offered meals to patients but not their caregivers, which increases expenses during hospitalization. Moreover, most of the caregivers were unable to work; some lost their jobs and relied on a few donations from their friends and relatives which worsened their financial burden. These findings are in agreement with other studies that describe the financial burden of care giving and catastrophic spending associated with a cancer diagnosis [38, 39]. Caregivers also reported lack of time to interact with their friends and misunderstandings in the family brought about by the care giving burdens and responsibilities, although some reported reconciliations brought by the need for collaborative efforts to support the patient. These study findings are supported by Van Roij and colleagues in their exploration of the social consequences of advanced cancer among patients and their FCGs reporting positive and negative shifts in the quantity and quality of their social relations [40].

This study also highlights hospital tensions experienced by FCGs such as long waiting time, overcrowding, insecurity, poor sanitation and few health workers to attend to their concerns. Participants were concerned with the long waiting time between referral time and the first doctor-patient interaction which delays diagnosis and treatment contributing to poor prognosis. The UCI is the only tertiary cancer facility in the country receiving patients from all regions of Uganda in addition to those from neighboring countries, as such; it serves a wide geographic area which explains the long waiting time. Despite efforts to decentralize cancer care to some regional referral hospitals in the country, there are still challenges of diagnostics, radiotherapy, and human resource in these facilities which makes patients to opt for UCI, further worsening its patient load.

Despite multiple challenges of care giving, FCGs remained motivated by the financial and emotional support they got from friends, relatives, health workers and the community. In addition, FCGs viewed care giving as a form of reciprocity and giving back the love and care they received from their loved ones before the illness which is a major source of motivation. Care giving is taken as a socio-cultural obligation which is deeply rooted within one’s cultural sub consciousness. That is, family members take on the caring role as an automatic duty that arises naturally without conscious thought to support a family member with a terminal illness like cancer [41]. For this reason, FCGs may take caring burdens and challenges as part of the role and persevere until the patient cures or succumbs to the disease. Nevertheless, the health system needs to structure interventions to motivate and support FCGs in their role.

### Study limitations

The limitations of our study include; (i) we recruited caregivers of patients with advanced cancers, hence these findings may not apply to caregivers of patients with early-stage cancer. We recommend future researchers to include caregivers of all stages and possibly cancer specific surveys so that we can easily know how the experience changes over the course of the disease; (ii) we also conducted at a single site in the country’s capital. Studies at multiple and regional cancer care facilities are needed to allow a comprehensive understanding and comparison of care giving experiences between caregivers in the urban and those in the rural setting; (iii) we recruited participants who were able to speak either English or Luganda, leaving out experiences of individuals who could not speak either language, yet Uganda is a multicultural and multilingual nation. Future researchers need to broaden language selection to reduce this selection bias; (iv) elderly FCGs were not represented in our study despite their unique role in care giving. They are known to experience multiple care giving effects (psychological, physical, and somatic symptoms), and have a strong connection to the cultural norm. Their experience and how possibly they cope should be investigated by future researchers; (v) the study team involved members of the UCI which may have affected the interpretation of our study findings. However, we included members from other sectors and perspectives to reduce on such bias; (vi) we excluded caregivers who were physically ill or weak to complete the interview yet this group of FCGs could have experienced more severe burden of care giving; and lastly (vii) we did not capture information on the patients treatment modalities which could have affected FCGs experiences.

Despite these limitations, this study is the first explorative qualitative study to describe care giving experiences of family caregivers of patients with advanced cancer in Uganda. In addition, we recruited a diverse sample with a wide range of characteristics such as age, gender, relationship with the patient, employment status and duration of caring. This enabled us to fully explore family caregivers’ experiences with diverse backgrounds. Moreover, study was conducted at the national referral cancer care facility which gave us access to caregivers from different parts of the country.

## Conclusion

This study illuminates the care giving experiences of family caregivers of patients with advanced cancer in Uganda. Through narratives of FCGs, it has been shown that FCGs play a fundamental role in the care of patients with advanced cancer throughout the cancer trajectory. This care giving roles affects all dimensions of their wellbeing; they experience physical symptoms, psychological distress, and financial drain, social, spiritual and cultural problems that need special attention. Furthermore, FCGs highlighted role conflict and health system tensions but also identified sources of motivation and support as friends, relatives and the community as a whole; highlighting financial support as the main support need to improve the care giving experiences.

These findings have significant implications for policy interventions. First, there is a need to further decentralize cancer care and strengthening the available regional cancer centers. This will cut financial costs, improve social roles and reduce role conflict. Second, the national cancer care policy should integrate psychological care of FCGs among services provided at the facility to reduce mental health symptoms in this vulnerable population. Third, the government should strengthen the home-based palliative care model with support programs to assist caregivers in their caring role such as provision of means of transport for patients and their caregivers to health facilities when need arises.

## Supporting information

S1 File Interview guide. (DOCX)

S2 File Supporting data. (DOCX)
