# Supplementary material for: The caring experiences of family caregivers for patients with advanced cancer in Uganda: A qualitative study
Source: PLoS One. 2023 Oct 25;18(10):e0293109. doi: 10.1371/journal.pone.0293109 (PMC10599584; doi:10.1371/journal.pone.0293109)
Supplement: S1 File — (DOCX) [file pone.0293109.s001.docx]

**Interview guide**

| - Please explain how you got to know that your patient has cancer? - Tell me about your caring journey to Uganda Cancer Institute - Could you please tell me your experience in taking care of your loved one? (Relationship with the patient, duration, actual experience) - Could you please explain how the care giving role has impacted your life? (Physically, psychologically/emotionally, socio-economically, spiritually, culturally) - How has the care giving role affected your relationships (family, friends, relatives etc) - How has your employment been affected? - Could you please tell me how the care giving process has changed your life?   (Could you please tell us about your typical day and how it has changed?)   - In relation to patient’s activities? - In relation to your activities? - What do you find difficult/challenging in the care giving process? Please explain?   Financial? Transportation? Health system-related? Hospital conditions /personnel?   - What do you find rewarding in the care giving process, please explain? - What do you gain from the care giving experience? Please explain? - What lessons have you learnt in the care giving process? Please explain? - Where do you get support from as a caregiver? Please explain   Family? Community? Health workers?   - What is your motivation to take care of your loved one? - What keeps you going? Please explain? - Which area do you think you need more support? Please explain - Is there something else you would like to discuss? |
| --- |
