# Supplementary material for: The caring experiences of family caregivers for patients with advanced cancer in Uganda: A qualitative study
Source: PLoS One. 2023 Oct 25;18(10):e0293109. doi: 10.1371/journal.pone.0293109 (PMC10599584; doi:10.1371/journal.pone.0293109)
Supplement: S2 File — (DOCX) [file pone.0293109.s002.docx]

**SUPPORTING DATA (INITIAL CODES)**

Initially doubts that the patient has cancer

Local doctor refers patient to UCI for further management

Cancer symptoms treated with local herbs

Describes cancer symptoms

A learned relative directs caregiver to UCI

Smooth and friendly admission process

Feels happy when discharged because he is able to go to work and put it in order

The workplace becomes disorganized when he is away in the hospital

Fails to monitor/supervise his business hence his employees cheat him

Has no control over his daily routine while in the hospital,

Communicates patients’ condition to doctors.

Washes patients clothes, brings food, buys drugs, feeds patient and administers drugs

Caregiving is hard in the beginning but it gets easier

Has no control over his daily routine while in the hospital,

Cannot do anything for himself while in the hospital

Lacks money to buy drugs, food and transport

Calls relatives for help with money for transport

Poor communication by health workers

Health workers delay to respond when called for help

Harsh communication by health workers

Feels that the patient is mistreated by the health workers

Asks money from people but they don’t have since they had not received their salaries yet

Shortage of doctors on weekends

Appreciates health workers when the patient gets better

Sleeping on the floor makes him fatigued

Dirty toilets that lack privacy

Lack of water in the toilets

Toilets misused by patients and caregivers

Afraid to use toilets, fears infections

Caregivers remind each other to pick patient’s medicine

Form of peer support

Sometimes misses appointments when he has no money for transport

Difficult to move from home area to the UCI, its far

Has learnt signs and symptoms of cancer

Plans to refer people with similar symptoms for medical check up

Finds it hard to move with the patient

Leaves patient with relatives in the city to look for money

Feels rewarded when he sees the patient alive

Has learnt to pray harder than before

Appreciates friend he and the patient have got in the hospital

Demoralized when he sees other patients die

Other caregivers look sad

Afraid that if he loses his job, caregiving will become more difficult

Would stop caring if he lost his job

Beginning of cancer symptoms

Long journey of caregiving involving travelling long distances, visiting many hospitals with periods of improvement and recurrence of symptoms before diagnosis

Cancer spreads to other areas of the body, patients’ condition worsens

Feels sad when the patient is not well

Feels chest pain, headache,worry, scared of patients death

Feels stressed when the situation worsens

No time for friends

Takes the patient to be her mother

Hard to go to church

Stressed when the patient is admitted during her exams

Helping the patient, bathe, feed, story telling

Patient doesn’t feel loved if she doesnt take care of her

Its hard when the patient refuses to eat

No money at home

Use ambulance for transport but its expensive

Money gets done

Health workers are not rude, they are social

Crowded wards

Shares hospital food with the patient

Feels good when the patient talks to her

Learnt how to give medication and how to remove the drip

Lack of patient education/communication

Patient skips to take medicine when the caretaker is not available

Politician helps them with money

Her love for the patient keeps her going

Cancer diagnosis made at a private hospital following longstanding symptoms

Caregiver is in and out of the hospital because the patient is oxygen dependent

Cancer first diagnosed as other diseases

Robbed while in the hospital, loses money, phones and medical records

Expensive and unavailable drugs

Affords only half the prescribed dose of drugs

Help from relatives

No time to check on people at home

No one to take care of children back home, neighbors help

Feels bad as she is unable to take care of her children

Believes god is present, taking care of the patient

The patient used to help her before the sickness. She feels she has to give back to him

Relatives help with financial support

Improved relationship within the family

Appreciates relatives for checking on them

Gets exhausted but cant give up

Stays motivated because she wants the patient to be better

Faith in God keeps her going

Distressed when the patient condition worsens. Can’t sleep

Crowded ward with no privacy

Little food provided by the hospital

The patient refuses to eat and she has to look for other types of food

Sacrificed to leave her children alone at home

No time to move out of the hospital

New life of sleeping in the hospital

Feeds the patient, washes, bathes

Transport means for the patient

Consoled by seeing other patients in a worse situation

Caregivers have to be understanding to the patients

Describes cancer as a complex disease

Some health workers are rude to patients

Nurse yells at a patient

Feeling of helplessness

Initially doctors don’t know what they are treating until proper investigations when a cancer diagnosis is made

Cancer treated as syphilis; investigations come out normal., caregivers wanted to know what he was suffering from

Rude statement from a doctor

Slept outside, hit by rain

Lack of money to afford medicines

Body pain and fall sick due to poor sleeping facilities

Feels sad when her husband complains

Feels abandoned by the relatives

Patient’s condition deteriorates

In good terms with God

Calls relatives to send some money

Hospital life is so stressing

Seeing the patient cry makes her feel sad

Helping the patient

Everything she does in the hospital is about the patient, none for herself

Bathing her mother is against her culture, it bothers her

Misconception about contracting cancer through contact with patients blood

No ppe, fears contracting hiv as she cares for the patient

Getting food is difficult yet the patient refuses it sometimes

Painful someone who used to eat properly but can no longer

Siblings help only sometimes

Language barrier

Health workers are good

Good communication with doctors

Poor sleeping facilities

Patients need patience

Patient gets tired of the hospital yet he is not well, this saddens the care giver

Learns to treat other people well

Becomes more faithful to god

Needs support with buying medicines

Needs support to carry the patient

Sold plot of land to keep patient in the hospital

Patient asks to go home because money is over

Trained about cancers

Thinks cancer came from a land wrangle

Feels bad, misunderstandings within the family

Cancer first treated as TB

He decides to have a second opinion from a senior doctor

Happy and relieved to know what the child was suffering from

Referred to Mulago by senior doctor

Did many investigations

Investigations are expensive

Hospital director reduces the price of investigations for him

No one to take care of things back home

Hospital director and friends help him with investigations

Feeding is very expensive, good quote

Medicines are expensive

Happy to know patients’ diagnosis

Relieved when patients condition improves

Travels from vey far, transport costs are high

Decides to stay in the hospital to catch up with hospital appointments since he comes from very far

No job anymore, its hard to provide for his family back home

Local herbs again, thinks the child was bewitched

His wife decided to also come to hospital

Rents a nearby hostel but it expensive

Decides to do manual work in order to manage hospital expenses

Sister helps him out

Neighbors tell him to use herbal medicine

Neighbors at home call and give advice, he appreciates

Unable to cultivate his garden, his animals were stolen because he was not around

His house collapsed because he was not around to innovate it

Chooses to take care of his child despite all hardships

Cancer affects other body parts, told the child will not survive

Afraid when told to take child home that he will not survive

Another doctor gives the child another chance

To advice anyone with similar symptoms to go to hospital immediately

Cleans patient and bedsheets, prepares food

Cant take care of his other problems

Cant but new clothes

Cant work when the child is admitted

Has to stay because the mother cant speak English

No money to have a balanced diet

Only one person is allowed to stay in the hospital yet he needs help

Sleeps for only 2hours in the night, no time to rest

Wonders if its okay for a doctor to tell a patient to go home and die

Fails to understand why a doctor tells another patient ot be taken home to die

Caregivers in shock, pain, crying when told to take patient home, that he wont survive

Health workers ignore their duties

Caregivers do what the health workers are supposed t do instead

Caregivers are ignored, they only take care of the patient

Little food that is not enough for both the patient and the caregiver

No one is available to be accountable for little food

No water for toilets

Old mattresses

Nurse refuses to give blood to the patient

Characters of health wotkers

Health workers ask for money from caregivers

Patient improvement is rewarding

Learns to be tolerant

Learns to feed the patient with a balanced diet

You don’t give up on seeking help for the patient

Asks the government to help the sick with money and other things

Asks for more mattresses

Advises that health workers should be trained about signs of cancer to allow a quick diagnosis

Child was healthy since birth

Symptoms of cancer started at 16 years

Symptoms progressed despite seeing a doctor

Child became paralysed with disability before a cancer diagnosis was made

Cancer diagnosis is made

Patients condition deteriorates and he is discharged but the caregiver refuses to take him home

Patients suffers end of life symptoms, wounds, nothing is being done in the hospital

Patient can eat and drink, there is some little improvement

Buys food for the patient

Turns, cleans the patient

Prays and fasts for his son

Difficult to leave the patient to go to work,

Employers don’t understan

Sometimes leaves the patient with aunt or brother

Juggles work with caregiving

Tired, stressed with so many responsibilities

Has another child at home with a chronic illness

Has to take care of other children

Employers are not understanding

No benefits of caregiving, everything hard

Feels stuck with so many responsibilities

Has to buy everything

Health workers are good but they hate wounds

Relatives help with money

Learnt how to wash wounds

Feels good with patients’ improvement

Patient condition improves

Church members visit and pray for the child

Doesn’t know what to do. She is just waiting and praying for the patient

Child was unwell since birth, diagnosed with cancer at 2years, sugery done at 3 years, moved from health center 4 through 5, 6, then antional referral

Child was readmitted for radiotherapy

Health workers explain to caregivers whatever they are going to do

Health workers do their best to treat the patient

Long time in the hospital, leaving other responsibilities at home

Many responsibilities but no money

You ask for money from friends who also have bigger problems

Home misses him as the head of family

Problem at work

Now depends on a small business managed by the wife

Thanks god for the patients condition is improving

Pleased with doctors for doing their job well

Thanks god for everything

Disagreements with the spouse

Feels despised in the family since he no longer earns and provides for the family

Feels despised in the family since he no longer earns and provides for the family

Feels irritated that he cannot execute his role as head of the family

Can’t afford to take his child to a better school

Unable to support his relatives he previously cared for

Feels like a burden to colleagues

Feels tat he just adds misery to his relatives

Improved his understanding of information

Improved his relationship with other people

Learnt to help people in need

Learns to be ready for any situation

Satisfied that he is doing his role very well

Parents discuss and cone to a conclusion that the father should stay with the child in the hospital whereas the mother should at home

They pray a lot

Washes patients’ clothes

Feeding the patient is most difficult

Frustrated when the patient gets other symptoms

Health system is tricky and complicated

Poor communication between health workers and patients

Takes long to be attended to following admission

Senior doctors listen more than young ones, but they are so busy and hard to reach

Unfortunate scenario at radiotherapy department

Investigations are scheduled for so long, caregivers afraid that the patient may not make it, and have no one to guide them

Failure to find a health worker to help you

Investigations are very expensive, feels like Mulago is for the rich, money first

A caregiver with language barrier is stranded with a sick child and cant see any health worker for a whole day

Wonders if anyone is concerned or accountable for the bad situation in Mulago

Long distances, high transport costs

Leaving from home for a long period, not knowing where he is going to sleep, or eat, no one to help you

Transport to mulago is hard, ambulances are expensive

Motivated when the patients condition improves

Encouraged by senior health workers

Junior health workers are not good

Happy to be a caregiver because he learns a lot

Learns to accommodate all sorts of people

Appreciates support from his wife

Encouraged by others to keep going

Believes that God will help him through this situation

Needs financial support

Asking himself why the government fails to decentralize cancer services

Urges health workers to advocate for their patients

Complains about the health system and the government

Appreciates investigators for carrying out research

Realizes that the patient has symptoms which are not relieved by medicine from a clinic

Diagnosed with cancer at a private clinic and referred to UCI

Expected an operation following referral to UCI

Expected to see the doctor immediately but was disappointed upon an a late appointment

Their reception at the UCI was brutal

Encouraged by the patient to be calm and patient with the receptionist

Afraid the patient may not make it to the appointment date, thinks about going to another country.

Makes sure transport means are available all day

Caregiver spends time to study the patients character in order to have an understanding relationship with him

Decided to suspend everything in order to take care of the patient

Thinks that the patient is elderly, delicate and needs full attention

Makes sure a car is available and he drives the patient to hospital

Does not delegate because the patient is emotional

He has to beg for mercy from his boss to understand that he is in the hospital

Caregiving is doing good, someone will return the favor

Realizes that he is at risk for cancer, and starts being careful

Wakes up so early

Financial needs are many

Siblings are jealous of him because he is close to their father

Family misunderstandings over the caregiving role

Abandoned the patient for his siblings and it dint end well

Patient collapses when he learns that the caregiver abandoned him

Has bult a good relationship with the patient

Describes a misunderstanding he got with the patient

Patient gives up on his life, he feels like he is going to die

Patient is tired of the hospital, curses his life, he is tired

He helps those with no caretakers, takes them home in his car, he realizes that people have broken families so no one can take care of them while in the hospital

He loves taking care of the patient, he is paying him back

Can’t make a formal program, uncertain about the next day

Patient blesses him when he prays for him

Caregivers have no program for the day

Can’t sleep, the patient’s needs require him awake

Wife understands he has to be way, but children miss him, he cant assure them of the day he will be back home

Does what is necessary at the moment

It stigmatizes the patient to use gloves while touching him

Carrying the patient is most challenging

Patient prefers that he takes care of him, no one else

Thinks that health workers are doing their best but patients are so many

Gate keepers are not understanding, they command a lot of authority

He works because he knows that God will reward him

Learns that one needs routine checkup after 45

Learns to be careful and avoid risky behaviours like smoking and taking alcohol

Patient regrets that if he knew about the disease earlier, he would have avoided it or come early for treatment

Agreed with his wife to build near the hospital to make caregiving easier

Family members and friends send them some money to use

Caregivers need psychologists to talk to them because some neglect their patients while others mistreat them

Patient refuses to go home following discharge because he fears to be mistreated from home

Health workers put their attention on only patients, caregivers are ignored

It takes so long to see the doctor following referral to UCI

Caregivers and patients are ignorant

Symptoms of cancer begin but the patient does not disclose to the caregiver

Patient was taken to the hospital when symptoms got worse

First taken at local clinic, referred to a RRH which suspects cancer

Investigations delayed since there was no money

Resorts to going to a nearby hospital that repeats similar tests, time is spent and it also refers them to another hospital which diagnoses cancer and refers then to the national hospital

Smoothly admitted at cancer institute

Earlier health workers did not fully disclose to the patient and caregiver, the diagnosis

counselled at uci, following disclosure of the diagnosis, the patient was depressed and in denial

Given an appointment to see the senior doctor, 2months ahead

Patient’s condition deteriorates before the appointment date

The tube could not be inserted within UCI and was unaffordable

A friend connects to them to a doctor in the hospital who does the procedure

Returns for the appointment, but the bag with appointment letter and personal property was stolen

Moving with a weak patient using public means is difficult

The taxi conductor didn’t want to take them, feared that the patient was too weak, and could die on the way

Felt like giving up on the patient because of the way they were treated buy the taxi driver

Has siblings but he caters for most of the needs in the hospital, he feels that he has to be around

Unable to be in the garden during planting season

Inability to work for him is a blessing in disguise, he has time to care to care for the patient

When discharged, the mother at home helps to feed the patient and prepare meals

His health is also negatively affected, he hardly gets time to eat yet he moves a lot

The patient feels loved and cared for otherwise he would have given up

Feels that patient is in endless pain, doesn’t know whether it will end

Feels that he has neglected his duties back at home

Keeps in touch with home via phone calls, he sends them some little money and directs and guides them on what to do

Used up all his savings, sold his cow in order to survive in the hospital

Helped by family members, church members and friends

Bath the patient, clean the bed, looks for his feeds, feed him, give him medication, massage him, wash his clothes, and keep around him

Moves the patient around, wait and picks results, buys drugs,

Traditionally not okay to bathe your parent

Long waiting time before you see the doctor

Expensive drugs

He wishes all services were available at UCI, moving a week patient is very tiring, the patient almost collapsed

Almost cried when the doctor said that he won’t see his patient following losing the file

Caregivers misuse the tolilets

Has got time to spend with his father, they chat, the father trusted him with his ATM card, something he couldn’t do before.

The patiet wouldn’t be alive if he was not taking care of him

Wife slaps his sick husband

One has to understand the situation and leave within his means

Hey have to take care of their father as a way to pay him back for educating them and taking care of them

Initially patient hides symptoms from care givers

Initial doctor found nothing was wrong with her, gave her some medicine and gave her a review date

Unable to access the hospital due to COVID-19 restrictions

Patient told that she is bewitched and starts taking herbal medicine, only for symptoms to worsen

Took patient to the hospital when symptoms worsened, a cancer diagnosis is made and she is refereed to UCI

School fees used for the patient

Unable to go to school for three weeks

Feeds the patient but she refuses to eat

Feeds the patient, washes clothes, buys medicine

Feels sad that her mother is in such a situation

Spends all the time with her mother, can’tvisit friends

Cannot go for internship like the colleagues because she has to take care of her mother

No one else can take care of the patient

Relatives view her as if she wants her mother to die, it is painful for her to process

Relatives refuse radiotherapy for the patient

Has no time to go to church to pray

Feels bad to bathe her mother but no one else can do it

Fears to get infected when washing clothes without gloves

Feeding is hard because she refuses to eat

Father sells his property to take care of the patient

Transport is difficult

Doctors treat them well

Caregivers knowledge about cancer as an incurable disease

She is paying for all the good things her mother did for her

She is so close to the patient, she is happy

Care giving has united the family

Has learnt that cancer has no cure

Seeing patients with a similar disease cure, motivates her as a caregiver, gives her hope that her mother will be okay

An organization helps them access blood

Appreciates the referring hospital for treating them well

Needs help convincing patient to go for radiotherapy

Pampers are expensive

Few beds for patients, sometimes patients sleep on the floor

Dirty toilets could infect patients and caregivers
